# The Prevalence of Thyroid Papillary Microcarcinoma in Patients With Benign Thyroid Fine Needle Aspiration

**DOI:** 10.7759/cureus.11820

**Published:** 2020-12-01

**Authors:** Abdullah H Alshathry, Nawaf Z Almeshari, Abdulaziz S Alarifi, Abdullah M Aleidy, Saleh Aldhahri

**Affiliations:** 1 Otolaryngology, College of Medicine, King Saud University, Riyadh, SAU; 2 Otolaryngology, King Saud University, Riyadh, SAU

**Keywords:** thyroid, microcarcinoma, ptc, fna, bethesda

## Abstract

Introduction

Thyroid nodules are a very common clinical finding in the general population. We use fine needle aspiration (FNA) as the gold standard workup test for a thyroid nodule, as it is capable of differentiating malignant nodules from benign in the majority of cases. Usually, FNA is done for nodules that are more than 1 cm; small malignant lesions that are less than 1 cm in size can be missed. That’s why the risk of having undiagnosed microcarcinomas in an otherwise benign FNA needs to be explored.

Aim

To estimate the prevalence of thyroid papillary microcarcinoma in patients with benign FNA and evaluate and correlate the FNA cytological results with the final histopathological diagnoses.

Methods

This was a retrospective study of 1543 post-thyroidectomy patients who underwent FNA cytology, were classified according to the Bethesda scoring system, and were admitted to two tertiary care hospitals in Riyadh, Saudi Arabia, from 2010 to 2019.

Results

Six-hundred-seven (607) out of 1543 FNA cytology results were reported as benign, 215 as malignant, and 73 as suspicious of malignancy. On final histopathology diagnosis, 81/607 (13.34%) of benign cases and 35/215 (16.28%) of malignant cases did not meet the initial cytology and were confirmed as papillary microcarcinoma. In patients with microcarcinoma after initial benign FNA (89.2%) found to have benign multinodular changes, compared to only (31%) of initial malignant FNA patients.

Conclusion

When non-surgical intervention is chosen in patients with benign FNA, the possibility of coexisting microcarcinoma with its variable prognosis should be taken into account and explained to the patient.

## Introduction

Thyroid nodules are a very common finding in clinical practice with a prevalence reported between 4% and 7% in the general population [1-2]. The ultimate goal when evaluating a patient with a thyroid nodule is to differentiate between benign and malignant lesions or at least to estimate the risk of malignancy in the existing nodule. According to the National Institute of Health (NIH), around 1.3% of the general population at some point in their lives will be diagnosed with thyroid gland cancer [3]. This high percentage emphasizes the need for the appropriate utilization and proper interpretation of the available diagnostic tests to achieve accurate and early diagnosis and to have the best possible outcome. Since its introduction to the medical field, fine needle aspiration (FNA) has proven itself to be the mainstay tool and the method of choice for the diagnosis of thyroid nodules [4]. FNA’s accuracy as a diagnostic tool combined with its simplicity, cost-effectiveness, and safe usage has made it the predominant tool for us. This simple diagnostic test complemented by other clinical and radiological findings guides the management of thyroid nodules, as it provides the necessary information regarding which patients will require surgical intervention from those who will not [5]. In terms of reporting the FNA results, Bethesda scoring system has been set as a standard way to classify the results according to cytopathologic features and the risk of malignancy: (1-Non diagnostic, 2-Benign, 3-Atypia/Follicular Lesion with Atypia of Undetermined Significance, 4-Follicular Neoplasm or Suspicious for a Follicular Neoplasm, 5- Suspicious of Malignancy, 6-Malignant) [6]. Because a thyroid FNA is usually done for the most suspicious nodule (based on the size and radiological features) and because the FNA inherited diagnostic limitation when done for nodules that are smaller than 1 cm, small malignant lesions that are less than 1 cm in size can be missed and only discovered after the surgical removal of the thyroid [7]. So, for better patient counseling, the risk of having undiagnosed microcarcinomas in an otherwise benign FNA needs to be explored.

## Materials and methods

A retrospective cohort study was conducted in two tertiary care hospitals in Riyadh, Saudi Arabia. Patients who underwent thyroid surgery with preoperative FNA between 2010 to 2019 were included. Patients who refused to do FNA and those who underwent the surgery without preoperative FNA were excluded from the study. There were 1543 thyroid surgeries performed with preoperative FNA during this period. All surgeries were done by head and neck consultants with more than 10 years of experience in thyroid surgery. The patient's FNA cytology was categorized according to the Bethesda reporting system into six categories (1-Non-diagnostic, 2-Benign, 3-Atypia/Follicular Lesion with Atypia of Undetermined significance, 4-Follicular Neoplasm or Suspicious for a Follicular Neoplasm, 5-Suspicious of Malignancy, 6-Malignant) [6]. The final histopathology was classified as “Benign,” “Papillary Microcarcinoma” if the largest malignant focus measures 1 cm or less, or “Malignant” for papillary carcinoma that measures more than 1 cm in the largest diameter or other thyroid malignancies [8].

The patient's medical data were collected using electronic charts in both hospitals. Demographic parameters, such as gender, body mass index (BMI), pre-operative thyroid-stimulating hormone (TSH), anti-thyroglobulin antibody, and anti-thyroid peroxidase antibody were evaluated. Data were entered and analyzed by using Stata program vision 15 (StataCorp LLC, College Station, Texas), simple descriptive statistics used to generate the mean, standard deviation, frequencies, and percentages. When relevant, the independent t-test and the chi-square test used to determine any statistical significance for continuous and nominal variables respectively. Anonymity and confidentiality were maintained in all phases of the study. This study has been approved by the institutional review board, research ethics committee in King Fahad Medical City.

## Results

The study included 1543 patients with 1242 females (80.49%) and 301 males (19.51%), with a mean age of 43.07 years for males and 40.6 for females. The BMI varies among the study group with a mean of 29.4 for males and 31.03 for females. Preoperative TSH and vitamin D values show no statistically significant difference between males and females (p=0.80) and (p=0.51), respectively. Out of a total of 830 patients, 38% had a positive preoperative anti-thyroglobulin antibody test; 84.2% of them were female with a p-value of 0.008. Moreover, 823 patients had done the anti-thyroid peroxidase antibody test, 29% were found to be positive, with 86.2% of them being female with a p-value of 0.005 (Table 1).

**Table 1:** Demographic data BMI (body mass index), anti-TG (anti-thyroglobulin antibody test), anti-TPO (anti-thyroid peroxidase antibody), preop (preoperative), P (p-value)

Parameter		Male (%)	Female (%)	Combined
Total number		301 (19.51)	1242 (80.49)	1543
Age *P=0.0037	Mean	43.07	40.6	41.11
BMI *P=0.0021		29.39	31.03	30.7
Preop TSH *P=0.8053		2.71	2.52	2.56
Preop vitamin D *P=0.5126		46.51	47.83	47.57
Positive Anti-TG *P=0.008		49 (5.9)	269 (32.41)	318 (38.31)
Positive Anti-TPO *P=0.005		34 (4.13)	211 (25.64)	245 (29.77)

**Figure 1:**
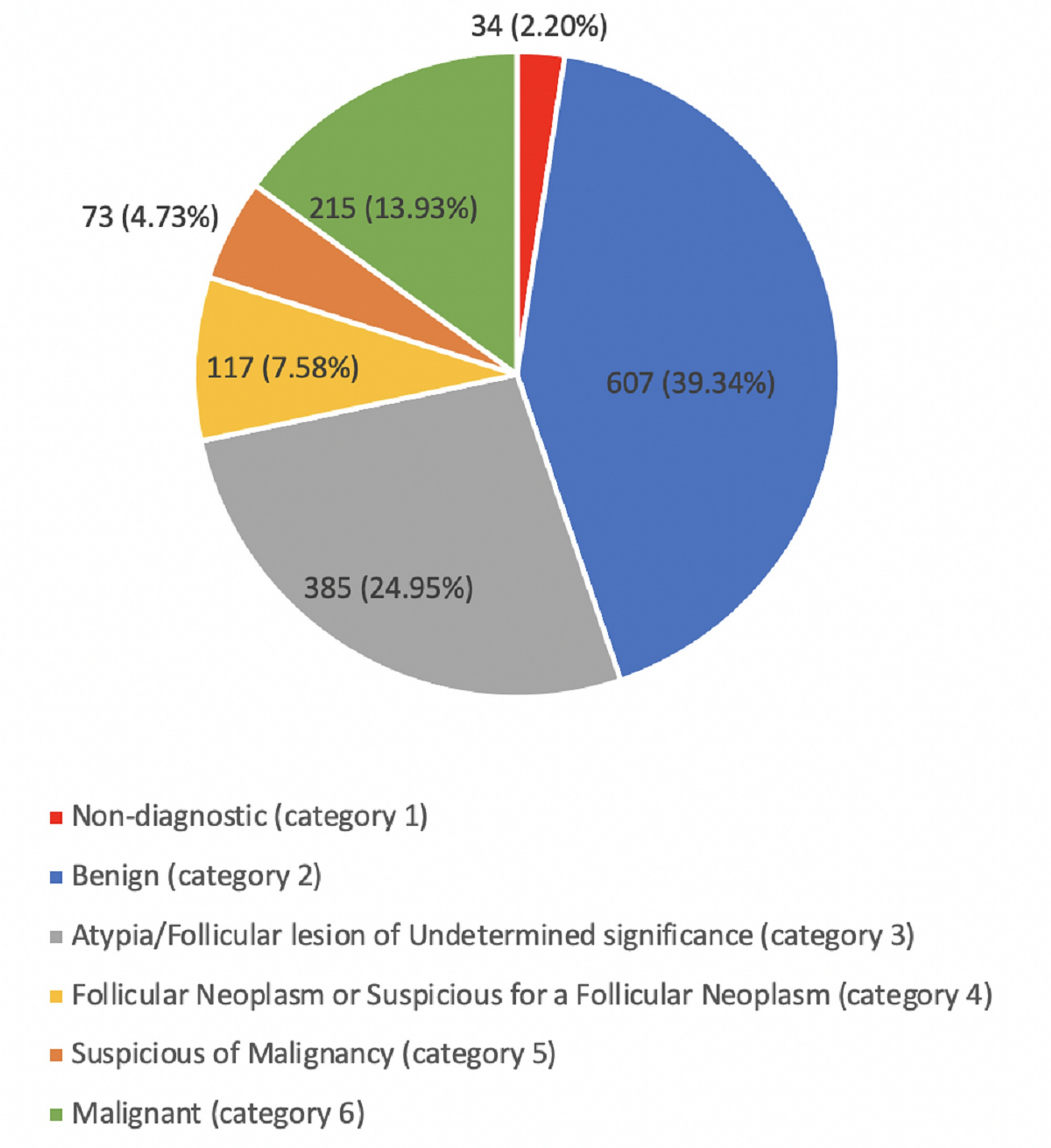
Distribution of FNA results based on the Bethesda scoring system FNA (fine needle aspiration)

The thyroid FNA results were categorized based on the Bethesda scoring system into six categories as follows (Figure 1).

The final histopathologic diagnosis showed 823 patients with benign pathology, 470 patients with malignant pathology, and 250 patients with papillary microcarcinoma. Out of 607 patients with initially benign FNA, 76 (12.52%) final histopathologic examination came back as malignant tumor and 81(13.34%) patients were diagnosed with papillary microcarcinoma. On the other hand, out of 215 patients with initially malignant FNA, the final pathology of 175 (81.40%) matched their initial result and 35 cases (16.28%) were diagnosed as microcarcinoma. The malignancy rates in the remaining categories can be found in Table 2. More details regarding the final pathology can be found in Table 3.

**Table 2:** Thyroid FNA and final pathology outcome. FNA code: 0 (not done), 1 (non-diagnostics), 2 (benign), 3 (atypia/follicular lesion of undetermined significance), 4 (follicular neoplasm or suspicious for a follicular neoplasm), 5 (suspicious of malignancy), 6 (malignant) FNA (fine needle aspiration)

	Final Pathology			
FNA code	Benign	Malignant	Microcarcinoma	Total
0	66 (58.93%)	29 (25.89%)	17 (15.18%)	112 (100%)
1	25 (73.53%)	7 (20.59%)	2 (5.88%)	34 (100%)
2	450 (74.14%)	76 (12.52%)	81 (13.84%)	607 (100%)
3	214 (55.58%)	96 (24.94%)	75 (19.48%)	385 (100%)
4	54 (46.15%)	41 (35.04%)	22 (18.80%)	117 (100%)
5	9 (12.33%)	46 (63.01%)	18 (24.66%)	73 (100%)
6	5 (2.33%)	175 (81.40%)	35 (16.28%)	215 (100%)
Total	823 (53.34%)	470 (30.46%)	250 (16.20%)	1543 (100%)

**Table 3:** Final pathology PTC (papillary thyroid carcinoma), FTC (follicular thyroid carcinoma), NIFTP (non-invasive follicular thyroid neoplasm with papillary-like nuclear features), Cancer (Ca)

Pathology	Number of cases	%
Benign pathology	783	50.75
PTC	402	26.16
Micro PTC	250	16.07
FTC	26	1.69
NIFTP	22	1.43
Follicular lesion of uncertain malignant potential	18	1.17
Minimally Invasive FTC / Hurthle cell Ca minimally invasive	16	1.04
Multiple types of thyroid Ca	6	0.38
Hurthle cell Ca	5	0.32
Medullary thyroid Ca	5	0.32
Anaplastic thyroid Ca	4	0.26
Lymphoma	4	0.26
Others	2	0.12
Total	1,543	100.00

In patients with benign FNA who had a microcarcinoma, 89.2% of them have benign multinodular changes in the thyroid histopathology in comparison to only 31% in patients with malignant FNA who turned out to have only a microcarcinoma in the final pathology.

Seven-hundred twenty (720; 46.66%) patients turned out to have a malignant tumor in the final pathology; papillary thyroid carcinoma was the most common malignant tumor (55.83%), followed by papillary microcarcinoma (34.72%). The others are listed in Table 3.

When evaluating the disease multicentricity in patients with benign FNA, there was no significant difference between patients with microcarcinoma and patients with a larger malignant lesion (31.7% vs 36.7%, respectively, with P=0.45). 

Considering a microcarcinoma as an incidental finding in the final pathology (because performing FNA for less than 1 cm nodules is not generally recommended) [7], the sensitivity and specificity of FNA in detecting malignancy in thyroid nodules (considering only Bethesda scores 2 and 6) are 69.72% and 92.99%, respectively. The false-positive rate was 7% and the false-negative rate was 30.27%. The positive predictive value was 81.39% and the negative predictive value was 87.47%. The accuracy of FNA in differentiating benign from malignant thyroid lesions was 85.88%.

After we correlated the results of the FNA and the final histopathological examination based on gender, we found that 9.80% of male patients with an initially benign FNA were diagnosed as malignant with 13.86% of females with benign initial FNA turning out to have a malignant tumor. However, using only the 2nd and 6th Bethesda categories, the sensitivity was higher in the male group (83.33%) than in the female (64.10%) while specificity, positive predictive value, and negative predictive value showed almost no difference between both genders.

## Discussion

In 1960, FNA was introduced in Sweden as the initial diagnostic test to categorize thyroid nodules due to its simplicity and cost-effectiveness [9]. However, there have been some variations in the diagnostic yields of FNA from one institution to another. Several factors can be considered as a reason for this variation such as the experience of the technologist, whether it was done under ultrasound guidance or not, the accuracy of interpretation of the final cytological results, including the classification of suspicious lesions, and the reporting system of the thyroid cytology [10-11].

As expected, our series were predominantly female (female to male; 4:1) with a comparable mean of age, which is in keeping with other studies [12]. Moreover, other parameters, including (BMI, Vit D, pre-op TSH, anti-thyroglobulin antibody, and anti-thyroid peroxidase antibody) showed almost no significant differences between both genders' groups.

In our study, 39.34% had an initially benign FNA, which is near what was reported by Lew et al. (32% out of 797 [13]. However other studies have shown large variability in the rate of benign FNA, 59% by Machala et al. and 14% by Wu et al. [12,14]. On the other hand, malignant FNA and those suspicious for malignancy cases in our study represent 18.66%, similar to a study conducted in Poland with 15.2% out of 1262 cases reported to be malignant [12]. However, different percentages of malignant FNA were found in different publications where they were reported to be as low as 5.3% and 6.8% [14,15]. This variability in the benign and malignant FNA rate can be attributed to the difference between the studied population. It did not surprise us that papillary thyroid carcinoma (PTC) and micro PTC were the most common malignant tumors, as that was in keeping with other studies [16-17]. 

Currently, thyroid cancer is considered the fastest growing cancer in the world, mostly due to the increasing number of incidental findings of papillary microcarcinoma in an otherwise benign thyroid [18]. Papillary thyroid microcarcinoma is defined by the World Health Organization (WHO) as a subtype of papillary thyroid cancer measuring 1 cm or less in size on the largest dimension [8]. Despite this increasing incidence, American Thyroid Association guidelines recommend not to do thyroid FNA in nodules less than 1 cm if they have a very low suspicion ultrasound (US) pattern [7].

Recently, different studies showed an increasing prevalence of microcarcinoma. In our series, almost one-third of the cases of thyroid cancers were confirmed as micro PTC. A meta-analysis conducted in 2008 showed that an average of 22.9% of malignant tumors are micro PTC [19]. Although more recent studies, such as the one reported by Louise Davies et al., showed a more comparable rate with ours (39%) [20].

These percentages of papillary microcarcinoma are considered high, especially if they can be found in good numbers in patients with benign cytology, as in our study (13.34%). The main reason for missing these malignant changes in benign FNA is the fact that the biopsy usually directs toward the largest/most suspicious nodule in US that is more than 1 cm in size according to the guidelines [7]. Moreover, in 59.6% of our patients with microcarcinoma, their nodule size was 5 mm or less, which makes it even more difficult to biopsy preoperatively. Several other factors could be responsible; one of these could be the presence of benign multinodular changes in almost one-third of patients with benign FNA with micro PTC compared to only 4% to those with larger than 1 cm malignant tumor. Having only one focus of micro PTC is another factor that may make the preoperative diagnosis even more difficult. Of the micro PTC, 67.13% has only a single focus in a multinodular goiter.

Although the prognosis for papillary microcarcinoma is better than other, large, well-differentiated thyroid cancer [21-22], we still do not know whether this better outcome is related to the nature of the disease or it is the early discovery. In general, unifocal micro PTC carries an excellent prognosis, however, multifocality, size above 6 mm, and age have been recognized as some prognostic factors that can alter this excellent outcome. Unfortunately, due to the excellent outcome of thyroid cancers in general, and the long follow-up needed to assess the impact of any intervention on prognosis, it is extremely difficult to do clinical trials assessing the outcome based on the intervention [23-25].

The reported sensitivity and specificity of thyroid FNA were 69.72% and 92.99%, respectively. This shows that the FNA has a moderate satisfactory ability in detecting thyroid malignancy in our study. However, many studies in the literature show values of sensitivity as low as 55.3%, 60.28%, and 61.53% [10,12,26]. According to the literature, the causes of this low sensitivity may be attributed to the use of US guidance, variability in the operator techniques and expertise, and the classification of undetermined and suspicious lesions [27-28]. On the other hand, higher ranges of sensitivity have been reported with 82.14% and 89.5%, considering that both of these studies had a smaller sample size with 122 and 724 cases, respectively [29-30]. Specificity reported in similar studies in the literature ranges from 74.9% [30] to 98% [17]. The diagnostic accuracy of FNA to detect malignancy for thyroid lesions is 85.88%. Other studies reported 83.6% [29] to 89.46% [12] accuracy of FNA, which supports ours.

## Conclusions

FNA cytologic examination of the thyroid nodule is a reliable diagnostic tool that carries high sensitivity and specificity. However, when non-surgical intervention is chosen in patients with benign FNA, the possibility of coexisting microcarcinoma with its variable prognosis should be taken into account and explained to the patient.
